# The effects of thrombocytopenia, type 2 diabetes mellitus, and endothelial dysfunction on clinical outcomes in patients with COVID-19

**DOI:** 10.5339/qmj.2023.3

**Published:** 2022-12-27

**Authors:** Mohanad Faisal, Fatima Alzahraa Al-Hattab, Aisha Mohammed Al-boinin, Bara Mahmoud Al-Qudah, Muhammad Aamir Waheed, Mohammed Danjuma

**Affiliations:** ^1^Hamad Medical Corporation, Doha, Qatar. E-mail: MFaisal2@hamad.qa ORCID: E-mail: https://orcid.org/0000-0002-2086-2957

**Keywords:** COVID-19, Diabetes Mellitus, Endothelial Dysfunction, thrombocytopenia

## Abstract

Diabetes mellitus is a well-recognized contributor to increased COVID-19 severity. Endothelial dysfunction has been implicated in the pathogenesis of COVID-19, while thrombocytopenia has been identified as a potential risk factor for severe COVID-19. In this study, we evaluated the combined effect of thrombocytopenia and other markers of endothelial dysfunction on disease outcomes in patients with type 2 diabetes and active COVID-19 infection. Our aim was to risk stratify patients with COVID-19 and type 2 diabetes mellitus, which can help identify patients with high-risk features who will benefit the most from hospital admission and a high level of care. This cross-sectional study was performed after reviewing secondary data of 932 patients with COVID-19 and type 2 diabetes mellitus in the outpatient and inpatient settings across Qatar between March 1, 2020 and May 7, 2020. Univariate and multivariate analyses, with adjustment for low platelet counts, were performed for the following variables: age, hemoglobin, white blood cells (WBC), lymphocytes, monocytes, eosinophils, alanine aminotransferase, aspartate aminotransferase, alkaline phosphatase, ferritin, D-dimer, and interleukin 6. Increasing age was associated with an increased risk for death and/or intensive care unit admission in diabetic patients with COVID-19 who have low platelet counts. These findings support the evidence found in the literature and give special attention to COVID-19 patients with low platelet counts and diabetes mellites. These results can guide physicians in making clinical decisions regarding hospital admission and escalation of care during follow-up in this population of patients.

## Introduction

Endothelial dysfunction, widespread inflammatory response, thrombopathy, and microvascular occlusion are among the proposed pathophysiological components of severe COVID-19 infection.^1^ Thrombosis and endothelial dysfunction are well-known pathways contributing to clinical deterioration in COVID-19 patients.^2^ In a case series from New York, one-third of COVID-19 patients with electrocardiographic signs of active ischemia showed non-obstructive coronary artery disease upon further evaluation, suggesting that microvascular dysfunction could be a likely cause of ischemia.^3^ In severe COVID-19 patients, markers of endothelial and platelet activation are increased compared to non-severe COVID-19 patients.^4^ Extensive microthrombosis promoted and aggravated by endothelial dysfunction could explain the profound D-dimer elevation and thrombocytopenia in severe COVID-19.^5^ Infection of endothelial cells by severe acute respiratory syndrome coronavirus 2 (SARS-CoV-2) results in endothelial activation and dysfunction. These result in elevated levels of pro-inflammatory cytokines such as interleukin-6 (IL-6). Higher levels of acute phase reactants (IL-6, C-reactive protein, and D-dimer) are also associated with COVID-19 infection.^6^ Serum ferritin levels were found to be an independent risk factor for severe COVID-19 infection.^7^

Endothelial dysfunction is also a common feature of the key comorbidities that increase the risk of severe COVID-19, such as hypertension, obesity, diabetes mellitus, coronary artery disease, and heart failure.^8^ Diabetes mellitus is one of the well-recognized comorbidities that lead to adverse clinical outcomes in patients with COVID-19.^9^

The effects of immune and non-immune thrombocytopenia in patients with COVID-19 have been extensively studied in the general population and found to result in adverse outcomes.^10^ The hypothesis of the combined effect of thrombocytopenia and markers of endothelial dysfunction and inflammation on the clinical outcomes of type 2 diabetic patients with COVID-19 remained unexplored till now. Examination of these data will provide clinical evidence for the multiplicative effects of these factors resulting in adverse outcomes in severe COVID-19. Such evidence will help risk stratify type 2 diabetes patients more likely to develop COVID-19 complications. This approach can identify patients more likely to benefit from direct interventions and prioritize hospitalization or escalation of care in patients with adverse laboratory features.

## Methodology

This is a retrospective cross-sectional study design that reviewed the secondary data of 932 type 2 diabetic patients. They were managed as COVID-19 cases in the outpatient or inpatient settings in the state of Qatar between 1/3/2020 and 7/5/2020. The study was conducted after receiving relevant approval from the medical research center. The sample size was calculated to give enough power to the study to minimize type 1 and type 2 errors. The primary study outcome was the composite of death and/or ICU admission in the study population.

Relevant socio-demographic and laboratory parameters were abstracted from an online patient information management system (Cerner) into a Microsoft Excel data collection spreadsheet. Patients were eligible for enrollment in this study if they were over 18 years old with type 2 diabetes mellitus and a confirmed diagnosis of COVID-19 (through a positive nasopharyngeal polymerase chain reaction swab). Data extracted included age, sex, complete blood count, coagulation profile, liver function tests (LFTs), D-dimer, ferritin, and IL-6. Patient outcomes after COVID-19 diagnosis, whether discharged without ICU admission, needed ICU admission, or died, were recorded. Patients were managed according to local COVID-19 guidelines for inpatients or outpatients; therefore, not all the parameters of interest in our study were available for each patient. Platelet count was subcategorized into three categories according to platelet count percentile among the study population. The first category was defined as patients with platelet counts < 185 cells/μl; the second category was patients with platelet counts between 185–232 cells/μl; and the third category was patients with platelet counts between 233–293 cells/μl. A univariate analysis was conducted on the following variables: age, hemoglobin, white blood cells (WBC), lymphocytes, monocytes, eosinophil, alanine aminotransferase (ALT), aspartate aminotransferase (AST), alkaline phosphatase (ALP), D-dimer, ferritin, and IL-6. Multivariate analysis, which was adjusted for patients with a lower percentile platelet count, was conducted on the variables which showed statistical significance in the univariate analysis, including age, chronic comorbidities, WBC, lymphocytes, monocytes, hemoglobin, and ferritin. Ethics approval was obtained from the independent review board (IRP) of the Medical Research Center (MRC) in Qatar with MRC number (MRC-01-21-167| Amendment - 01). Patient data were handled with confidentiality according to MRC guidelines.

### Data analysis

Continuous variables were expressed as mean ( ±  SD) or median (interquartile range), while categorical variables were expressed as counts (percentages). All study subjects were classified into quartiles of blood platelet levels, with baseline characteristics including socio-demographic parameters compared among these quartiles. The comparative means and medians of the various study covariates were estimated. Spearman's correlation coefficients were derived for the various study covariates. Categorical and ordinal logistic regression analyses were used to estimate the risk of clinical outcomes associated with percentiles of serum platelet levels. The estimated odds ratios (ORs) and 95% confidence intervals (CIs) for higher quartiles were compared to the lowest quartile. Unadjusted estimates of the relationship between various study covariates and lower percentile platelet count were derived through bivariate regression analyses. Variables with a *P*-value < 0.2 were then entered into multivariate regression models. The covariates included in the multivariable models were age, serum ferritin, WBC, neutrophils, lymphocytes, monocytes, eosinophils, and hemoglobin.

Further sensitivity analyses were carried out to ascertain the robustness of our findings. As previous studies have shown the predictive potential of endothelial dysfunction markers in identifying patients with COVID-19 who could potentially require ICU admission, multivariate analyses with and without these markers were performed to ascertain any shift in our point estimate. All analyses were performed using Stata (StataCorp. 2021. Stata Statistical Software: Release 17. College Station, TX: StataCorp LLC).

## Results

A summary of the socio-demographic and laboratory characteristics of the study population is shown in Table 1. There were 932 patients recruited, with a male-to-female ratio of 6:1. Of these patients, 236 were in the lower quartile of platelet counts with a median age of 52.144 ( ± 10.605) years. The primary outcome, which was the composite of death and/or ICU admission, was 10.3% in the study population (96/932), with 13 events among females (6 deaths and 13 ICU admissions) and 83 events among males (12 deaths and 83 ICU admissions). The mortality rate was 1.931% (18/932), while the other patients were successfully discharged. The rate of ICU admission was 10.3% (96/932).

Table 2 shows the outcome of the bivariate analysis of the study covariates. Advancing age was associated with a significant increase in the risk of mortality and/or ICU admission (OR: 1.11, CI: 1.054– 1.171, *p* < 0.05) in patients with low platelet counts. For the markers of inflammation and endothelial dysfunction, an increase in the risk of death and/or ICU admission in the setting of thrombocytopenia was associated with increasing serum ferritin (OR: 1.001, CI: 1.000–1.001, *p* < 0.000). A mild but significant increase in death and/or ICU admission with increasing ALT (OR: 1.004, CI: 1.007–1.00, *p* < 0.0006) and ALP (OR: 1.016, CI: 1.007–1.025, *p* = 0.001) levels were also observed in patients with low platelet count. In contrast, lymphocyte count (OR: 0.207, CI: 0.086–0.499, *p* < 0.000) and hemoglobin (OR: 0.577, CI: 0.471–0.70, *p* < 0.0008) were associated with decreased risk of death and/or ICU admission.

### Prediction of the risk of mortality/ICU admission and Thrombocytopenia

Table 3 shows the outcome of the multivariate analysis. Increasing age was consistently predictive of the risk of ICU admission in patients with lower platelet percentile (OR: 1.35, CI: 1.089–1.677, *p* = 0.006). Out of the three markers of endothelial dysfunction evaluated, only serum ferritin showed a trend toward predicting ICU admission or mortality in patients with lower platelet count (OR: 1.0007, CI: 0.9999–1.0016, *P* = 0.007).

## Discussion

Endothelial dysfunction is a principal determinant of microvascular dysfunction with subsequent organ ischemia, inflammation with associated tissue edema, and a pro-coagulant state.^11^ Pathology of endothelial cell dysfunction in COVID-19 patients’ autopsies showed the presence of viral elements within endothelial cells and an accumulation of inflammatory cells, with evidence of endothelial and inflammatory cell death. This could explain the systemic impaired microcirculatory function in different vascular beds and their clinical sequelae in patients with COVID-19.^12^

Ferritin plays an important role in intracellular iron storage. Serum ferritin is also widely recognized as an acute phase reactant and marker of acute and chronic inflammation. It is non-specifically elevated in a wide range of inflammatory conditions. Ferritin secretion is increased by iron and the cytokines interleukin-1-β (IL-1) and tumor necrosis factor-α (TNF-α) *in vitro*.^13^ In the study by Zhi Lin et al., serum ferritin was an independent risk factor for predicting COVID-19 severity.^7^

D-dimer is a fibrin degradation product released into the circulation following fibrinolysis of blood clots. It has two D fragments of the fibrin protein attached by a cross-link.^14^ Previous research suggested that high D-dimer levels could predict disease severity, lung complications, and thromboembolic events in COVID-19 even before they occur. A meta-analysis of 12 studies that included 2801 patients found a significant association between high D-dimer and increased severity of COVID-19.^15^ There is still controversy regarding the mechanism causing the increase in D-dimer levels. It is possible that this effect results only from an increase in fibrin formed in COVID-19 infection.^16^

Among the three chosen markers of endothelial dysfunction and inflammation, serum ferritin showed a trend toward predicting ICU admission or mortality in our study population (OR: 1.0004, CI: 1.000–1.001, *p* = 0.001). However, this trend was not observed in patients in the lower percentile of platelet count (OR 1.0007, CI: 0.9999–1.002, *p* = 0.007), which can be explained by a reduction in sample size. We attribute this lack of findings in these markers to a low number of observations for D-dimer and IL-6, as not all participants have undergone testing for these measures. Our study included hospitalized and non-hospitalized patients who were managed according to COVID-19 local guidelines during the first wave of COVID-19 in Qatar

Pre-existing endothelial dysfunction is observed in patients with diabetes, among other conditions. The US Centers for Disease Control and Prevention has reported that diabetes prevalence rises with increasing severity of COVID-19, from 6.4% in non-hospitalized patients to 24.2% and 32.4% in hospitalized and ICU patients, respectively.^17^ There is a bidirectional relationship between COVID-19 and diabetes mellitus. On the one hand, people with diabetes have a higher risk of developing complications when they present with COVID-19. On the other, the COVID-19 virus could act as a diabetogenic agent by binding to the angiotensin-converting enzyme 2 in pancreatic beta cells, causing acute dysfunction and changes in glucose homeostasis.^18^ Aberrant immune responses may contribute to disease progression by accelerating thrombotic and ischemic complications, leading to multiorgan failure and mortality. Our study was an attempt to risk stratify patients with diabetes using important socio-demographic and laboratory data. Identifying factors that predict complications of COVID-19 is pivotal for guiding clinical care, improving patient outcomes, and allocating scarce resources. Factors including age, comorbidities, immune response, radiographic findings, laboratory markers, and indicators of organ dysfunction may individually or collectively predict worse outcomes.^19^ In one of the first studies in Wuhan that studied COVID-19 disease severity, there was an association between clinical outcomes and multiple parameters, including age (r = 0.458, *p* < 0.001) and D-dimer (r = 0.477, *p* < 0.001).^20^ Our study supported this as we found that increasing age was associated with increased mortality and/or ICU admission among diabetic patients with low platelets.

It is evident that thrombocytopenia is associated with adverse outcomes. In a study of 167 confirmed cases of critically ill COVID-19, thrombocytopenia was associated with the deterioration of respiratory function. Low baseline platelet count was also associated with subsequent and long-term mortality.^21^ In another retrospective cohort study of 380 patients, thrombocytopenia was implicated in increasing mortality in patients with COVID-19.^22^ Our cross-sectional study was conducted because of the scarcity of literature on the prognosis of COVID-19 specific to patients with diabetes, thrombocytopenia, and elevated markers of endothelial dysfunction. To our knowledge, this study represents the first attempt at exploring the role of various thresholds of platelet counts in augmenting the risk of adverse outcomes in type 2 diabetic patients with COVID-19-related endothelial dysfunction. Consistent with earlier reports in high-risk COVID-19 patients, we found that advancing age consistently predicts the risk of ICU admissions among type 2 diabetic patients with thrombocytopenia. This undoubtedly provides an additional hematological marker for clinical risk stratification in these highly vulnerable patients. We also found that serum ferritin is a potential predictor of ICU admission and mortality in this population.

## Conclusion

In a cohort of patients with type 2 diabetes and COVID-19 clinical syndrome, thrombocytopenia in the setting of advancing age was associated with a multiplicative risk of both ICU admission and death. Study findings can be used in adjusting available prognostic scoring systems to increase the accuracy and validity of prognosis estimation. Furthermore, it can help guide hospital protocols for risk stratification of patients for admission, which is necessary for triaging COVID-19 patients who may develop severe disease.

### Future scope

Our study represents an attempt to stratify the risk of patients with diabetes mellitus who contract COVID-19. Our results demonstrate the increased risk of mortality and ICU admission in patients with low platelet counts and increasing age. Conducting a prospective study that will include diabetic patients and non-diabetic controls and require more standardized inclusion criteria, including relevant laboratory tests, would result in more robust outcomes.

## Ethical Statement

The authors confirm that they have no conflict of interest in conducting this study. Approval was obtained from the independent review board (IRP) of the Medical Research Center (MRC).

## Figures and Tables

**Table 1:** Baseline characteristics of the study population as a factor of quartiles of platelet levels of sample of diabetic COVID-19 patients. Data were collected between 1/3/2020 and 7/5/2020 at the start of the first COVID-19 wave.

Variables	Total number of observations	Quartiles of platelet counts (cells/μl)		
		< 185	186–232	233–293
**Patients**	932	236	462	234
**Age (years) Mean ( ± standard deviation)**	932	52.144 ( ± 10.605)	50.989 ( ± 10.646)	50.782 ( ± 9.920)
**AST^1^ Median interquartile range (IQR)**	887	35 (25,52)	25 (19,36)	25 (19,38)
**ALT^2^ median (IQR)**	912	32 (23, 53)	27 (19, 40.25)	28 (20, 49.75)
**ALP^3^ median (IQR)**	913	73 (60, 89.75)	74 (60.5, 91.8)	76.45 (62,92)
**PT^4^ median (IQR)**	290	12.2 (11.375, 13.4)	12.05 (11.175,13.125)	12.7 (11.3,14.025)
**APTT^5^ median (IQR)**	240	32.5 (30.125,35.125)	30.6 (28.7,33.4)	28.9 (26.7, 32.5)
Serum Ferritin median (IQR)	535	494 (278,864)	334 (154,548)	347 (101.15,723.2)
**Serum IL-6^6^ median (IQR)**	56	87 (58.5,852.5)	57 (21.5,161)	83 (54,313)
Serum D-dimer median (IQR)	218	0.59 (0.393,1.033)	0.56 (0.358, 0.948)	0.88 (0.405,2.015)
**Total WBC^7^ Median (IQR)**	929	5.4 (4.11,6.9)	6.8 (5.7,8)	7.8 (6.2,9.45)
**Lymphocytes (IQR)**	932	1.3 (0.955,1.788)	1.7 (1.3,2.4)	2.055 (1.4,2.6)

^1^ AST: Aspartate Transferase

^2^ ALT: Alanine Aminotransferase

^3^ ALP: Alkaline Phosphatase

^4^ PT: Prothrombin Time

^5^ APTT: Activated Partial Thromboplastin Time

^6^ IL-6: Interleukin 6

^7^ WBC: White Blood Cells

**Table 2:** Univariate analysis of the demographic and laboratory parameters in diabetic COVID-19 patients. Data was collected between 1/3/2020 and 7/5/2020 at the start of the first COVID-19 wave.

Value	Odds ratio	Standard error	z	P>I Z l^*^	95% confidence interval
**WBC^8^**	1.022	0.019	1.15	0.252	0.985–1.060
**Age**	1.111	0.030	3.92	0.000	1.054–1.171
**lymphocyte**	0.207	0.093	–3.51	0.000	0.086–0.499
**monocyte**	0.078	0.089	–2.23	0.026	0.008–0.732
**eosinophil**	0.087	0.206	–1.03	0.302	0.001–9.028
**hemoglobin**	0.577	0.060	–5.29	0.000	0.471–0.708
**platelets**	0.989	0.004	–2.74	0.006	0.981–0.997
**ferritin**	1.001	0.000	4.71	0.000	1.000–1.001
**AST^9^**	1.058	0.049	1.20	0.229	0.965–1.161
**ALT^10^**	1.003	0.001	3.50	0.000	1.001–1.006
**ALP^11^**	1.016	0.005	3.48	0.001	1.007–1.025
**IL-6^12^**	1.000	0.001	–0.32	0.749	0.998–1.002
**D-dimer**	1.007	0.049	0.14	0.892	0.915–1.108

*P-value was considered significant < 0.05,^8^ WBC: White Blood Cells,^9^ AST: Aspartate Transferase,^10^ ALT: Alanine Aminotransferase,^11^ ALP: Alkaline Phosphatase, ^12^ IL-6: Interleukin 6.

**Table 3:** Multivariate analysis adjusted to lower platelets count of sample of diabetic COVID-19 patients. Data was collected between March 1, 2020 and May 7, 2020 at the start of the first COVID-19 wave.

Value	Odds ratio	Standard error	z	P>I Z l	95% conf. interval	
**WBC^13^**	0.985	0.186	–0.08	0.935	0.679	1.42
**Age**	1.35	0.149	2.73	0.006	1.089	1.677
**Lymphocyte**	0.591	0.871	–0.36	0.721	0.033	10.622
**Monocyte**	4.190	8.384	0.72	0.474	0.083	211.541
**Eosinophil**	29.651	193.938	0.52	0.604	0.000	1.100
**Hemoglobin**	1.566	0.520	1.35	0.178	0.816	3.003
**Ferritin**	1.001	0.000	1.66	0.097	0.9999	1.002

^13^WBC: White Blood Cells
